# Impact of Sleep Deprivation on Cognition and Academic Scores: A Three-Month Longitudinal Study Among Indian Medical Students

**DOI:** 10.7759/cureus.91044

**Published:** 2025-08-26

**Authors:** Rohit Saroha, Soni Singh, Muneeb Kosvi, Nasar Ajmera, Prateek Grover

**Affiliations:** 1 Physiology, Santosh Deemed to be University, Ghaziabad, IND; 2 Physiology, Teerthanker Mahaveer University, Moradabad, IND; 3 Physiology, Lady Hardinge Medical College, New Delhi, IND

**Keywords:** academic performance, attention, chronotype, cognitive function, executive function, medical students, sleep deprivation, sleep hygiene, undergraduate medical education, working memory

## Abstract

Introduction: Sleep is essential for preserving cognitive function and academic achievement. Medical students who face significant academic pressures and lifestyle disruptions are particularly susceptible to sleep deprivation. Although there is increasing awareness of this issue, longitudinal research investigating its effects on cognition and academic performance, especially among Indian populations, remains scarce.

Objectives: This study aimed to evaluate the longitudinal impact of sleep deprivation on cognitive performance and academic achievement among first-year Indian medical undergraduates while controlling for significant behavioral and psychological confounding variables.

Methods: This longitudinal observational study, conducted over a period of three months, involved the enrollment of 80 first-year MBBS students (mean age of 19.1 ± 0.7 years), of whom 72 completed all assessments and were included in the final analysis. Sleep patterns were monitored daily using self-reported sleep logs. Cognitive function was assessed at baseline, six weeks, and 12 weeks using computerized tests for attention (Simple Reaction Time Test), working memory (Digit Span Test), and executive function (Stroop Test). Academic performance was evaluated based on internal assessments. Confounding variables, including screen time, stress, diet, and chronotype, were measured using validated instruments and accounted for in multivariable regression models. Statistical analyses were conducted using repeated measures analysis of variance (ANOVA), one-way ANOVA, Pearson correlations, and multivariable linear regression. All p-values refer to results from these tests unless otherwise noted.

Results: A significant decline in sleep duration was observed over time (from 6.8 ± 0.9 to 5.9 ± 1.1 hours, p < 0.001), accompanied by progressive deterioration in cognitive scores: reaction time worsened (p < 0.001), digit span declined (p = 0.002), and Stroop interference increased (p < 0.001). Academic performance scores demonstrated a positive correlation with sleep duration (r = 0.43, p = 0.001). Multivariable models identified sleep duration as an independent predictor of academic performance (β = +2.78, p = 0.003) and cognitive domains, after adjusting for all confounders.

Conclusion: Chronic sleep deprivation among medical students results in significant cognitive decline and diminished academic performance. The findings highlight the significance of implementing institutional sleep hygiene programs to maintain cognitive function and enhance academic performance among medical students.

## Introduction

Sleep is a fundamental physiological process that supports cognitive function, emotional stability, and memory consolidation [1]. Among university students, particularly those in the medical field, sleep deprivation has become a prevalent and persistent issue exacerbated by academic demands, irregular schedules, and psychosocial stressors [2]. While acute sleep restriction may have temporary effects, chronic deprivation is associated with impairments in attention, working memory, and executive functioning, which are critical domains essential for academic achievement [3]. The preclinical phase of medical education presents distinct challenges. Students are required to assimilate complex theoretical knowledge while concurrently adjusting to the demands of professional training. This transition frequently coincides with the development of unhealthy sleep patterns, which, if persistent, may undermine cognitive capacity and emotional resilience. The existing literature, predominantly cross-sectional in nature, has associated poor sleep quality with academic underperformance and psychological distress among medical students [4,5]. However, there is a paucity of longitudinal data, particularly from Indian cohorts [2,3]. In light of the increasing incidence of cognitive overload and burnout within Indian medical institutions [2,3], this study sought to examine the impact of progressive sleep deprivation on fundamental cognitive function and academic performance over time. Specifically, it investigates the progression of attention, working memory, and executive function over a three-month period in first-year MBBS students while controlling for potential confounding variables such as stress, screen time, dietary habits, and chronotype.

## Materials and methods

Study design and setting

This was a longitudinal, observational study conducted over a three-month period (July-September 2024) at the Department of Physiology, Santosh Deemed to be University, Ghaziabad, India. The study adhered to the Strengthening the Reporting of Observational Studies in Epidemiology (STROBE) guidelines for observational research [6] and received approval from the Institutional Ethics Committee (Ref. No. SU/R/2024/1353(1)).

Study population

Eighty first-year MBBS (Bachelor of Medicine and Bachelor of Surgery) students (aged 18-22 years; 40 male and 40 female) voluntarily participated after providing written informed consent. Recruitment occurred via an open invitation during student orientation.

Inclusion Criteria

The study included first-year MBBS students between the ages of 18 and 22 who had no previous diagnoses of neurological or sleep disorders and who agreed to participate in all evaluations by providing informed consent.

Exclusion Criteria

Students were excluded if they were taking medications that influence sleep or cognitive function, had a history of smoking, alcohol consumption, or substance abuse, had incomplete data at any point, or were found to have moderate-to-severe depression or anxiety during initial screening with the Depression Anxiety Stress Scale-21 (DASS-21) tool [7].

Sample size justification

The required sample size was calculated using G*Power 3.1 (Heinrich-Heine-Universität Düsseldorf, Düsseldorf, Germany), assuming a medium effect size (Cohen’s f = 0.25) for repeated-measures analysis of variance (ANOVA), 80% power, and alpha = 0.05. A total of 80 participants were enrolled, anticipating an approximate 10% dropout rate.

Sleep assessment

Participants maintained standardized daily sleep diaries, recording total sleep duration, sleep latency, and perceived sleep quality. Sleep quality was rated using a five-point Likert Scale (1 = very good, 5 = very poor), and weekly averages were computed for analysis.

Cognitive function assessment

Cognitive testing was conducted at three time points during the study: at baseline (Week 0), at the midpoint (Week 6), and at the endpoint (Week 12). Assessments were performed using a computerized battery developed with PsychoPy3, an open-source Python-based software (Open Science Tools Ltd., United Kingdom). Tests took place in a quiet laboratory setting under consistent environmental conditions. The Simple Reaction Time Test measured attentional efficiency by recording the time participants took to respond to a visual stimulus, with shorter reaction times indicating better attention [8]. The Digit Span Test, administered in forward and backward formats, required participants to recall digit sequences in order or reversed; the maximum correctly recalled sequence length was scored, with the backward format additionally assessing executive control [9]. Working memory was assessed using both Digit Span Forward and Digit Span Backward formats; however, in this study, only the combined total score was recorded and analyzed. The Stroop Color-Word Interference Test evaluated executive function by having participants name the ink color of congruent or incongruent words; performance was assessed using reaction times and errors, with an interference score reflecting inhibitory control [10].

**Figure 1:**
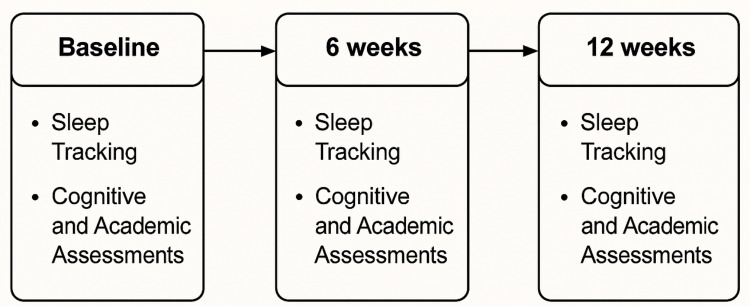
Timeline of sleep tracking and cognitive/academic assessments at baseline, six weeks, and 12 weeks

Figure 1 illustrates the assessment timeline across the 12-week study period.

Academic performance

Academic performance was assessed through continuous internal evaluations comprising multiple-choice question (MCQ)-based theory examinations, short-answer written tests, and practical examinations conducted via Objective Structured Practical Examinations (OSPE) in Physiology [11]. The composite academic score was calculated as the average percentage across all these components.

Assessment of confounding variables

To control behavioral and psychological confounders, validated instruments were administered at baseline, as summarized in Table 1. Psychological screening was performed using DASS-21, while perceived stress was measured using the 10-item Perceived Stress Scale (PSS-10) [12]. Chronotype classification was conducted using the Horne-Östberg Morningness-Eveningness Questionnaire (MEQ) [13,14]. Academic motivation was assessed using a structured Academic Motivation Profile Questionnaire (AMPQ), developed by the authors specifically for this research, based on established academic motivation constructs, and underwent pilot testing and internal consistency checks to ensure it captures relevant motivational domains among medical undergraduates. Dietary patterns were evaluated through a simplified semi-quantitative Food Frequency Questionnaire (FFQ), also developed by the authors for this study, based on previously validated food intake questionnaires, with validation steps including correlation analyses against dietary recalls or records, demonstrating acceptable validity for assessing dietary patterns in this population. The full versions of the AMPQ and FFQ are included in Appendix A and Appendix B, respectively, for transparency and replication. All instruments were used under academic fair-use policies.

**Table 1:** Instruments used for confounder assessment

Confounder domain	Instrument used	Timing
Screen time	Adolescent Media Practice Questionnaire (AMPQ)	Baseline
Diet and caffeine	Semi-quantitative Food Frequency Questionnaire (FFQ)	Baseline
Psychological stress	Perceived Stress Scale (PSS-10)	Baseline
Chronotype	Morningness-Eveningness Questionnaire (MEQ)	Baseline
Mental health	Depression, Anxiety, and Stress Scale (DASS-21)	Screening

Students with DASS-21 scores indicating moderate-to-severe symptoms were excluded from the adjusted statistical models. These variables were included as covariates in the multivariate analyses.

Statistical analyses

Data were analyzed using IBM SPSS Statistics v30 (IBM Corp., Armonk, USA) and R v4.3 (R Foundation for Statistical Computing, Vienna, Austria). Continuous variables were summarized using means ± SD. Repeated-measures ANOVA was used to evaluate within-subject changes across the three timepoints.

Modeling approach

The modeling approach involved initial Pearson correlation analyses to evaluate unadjusted bivariate associations. Multivariable linear regression models were then employed to predict academic and cognitive outcomes, with sleep duration as the primary predictor. Covariates included screen time, perceived stress score, dietary quality, and chronotype. Interaction effects, such as sleep × chronotype, were also tested but found to be statistically non-significant. Model assumptions were verified using the Shapiro-Wilk test for normality and variance inflation factors (VIF), with all VIF values below 2.1 indicating no multicollinearity. Statistical significance was defined as p < 0.05 (two-tailed).

## Results

A cohort of 80 first-year MBBS students was enrolled in this study. Of these participants, 72 (90%) successfully completed all assessments and were subsequently included in the final analysis. The mean age of the cohort was 19.1 ± 0.7 years, comprising 40 male and 32 female students.

Sleep pattern trends

A significant decline in sleep duration was observed across the study period, with all participants reporting lower sleep durations by the final assessment compared to baseline. At baseline (Week 0), students reported an average of 6.8 ± 0.9 hours/night, which decreased to 6.2 ± 1.0 hours by Week 6 and further to 5.9 ± 1.1 hours by Week 12 (p < 0.001, repeated measures ANOVA). This progressive reduction indicates cumulative sleep debt over the academic term. Repeated measures ANOVA revealed a statistically significant reduction across the time points (F (2,142) = 11.34, p < 0.001). Subsequent pairwise comparisons indicated a decline from baseline to both Week 6 (p = 0.03) and Week 12 (p < 0.001).

**Figure 2:**
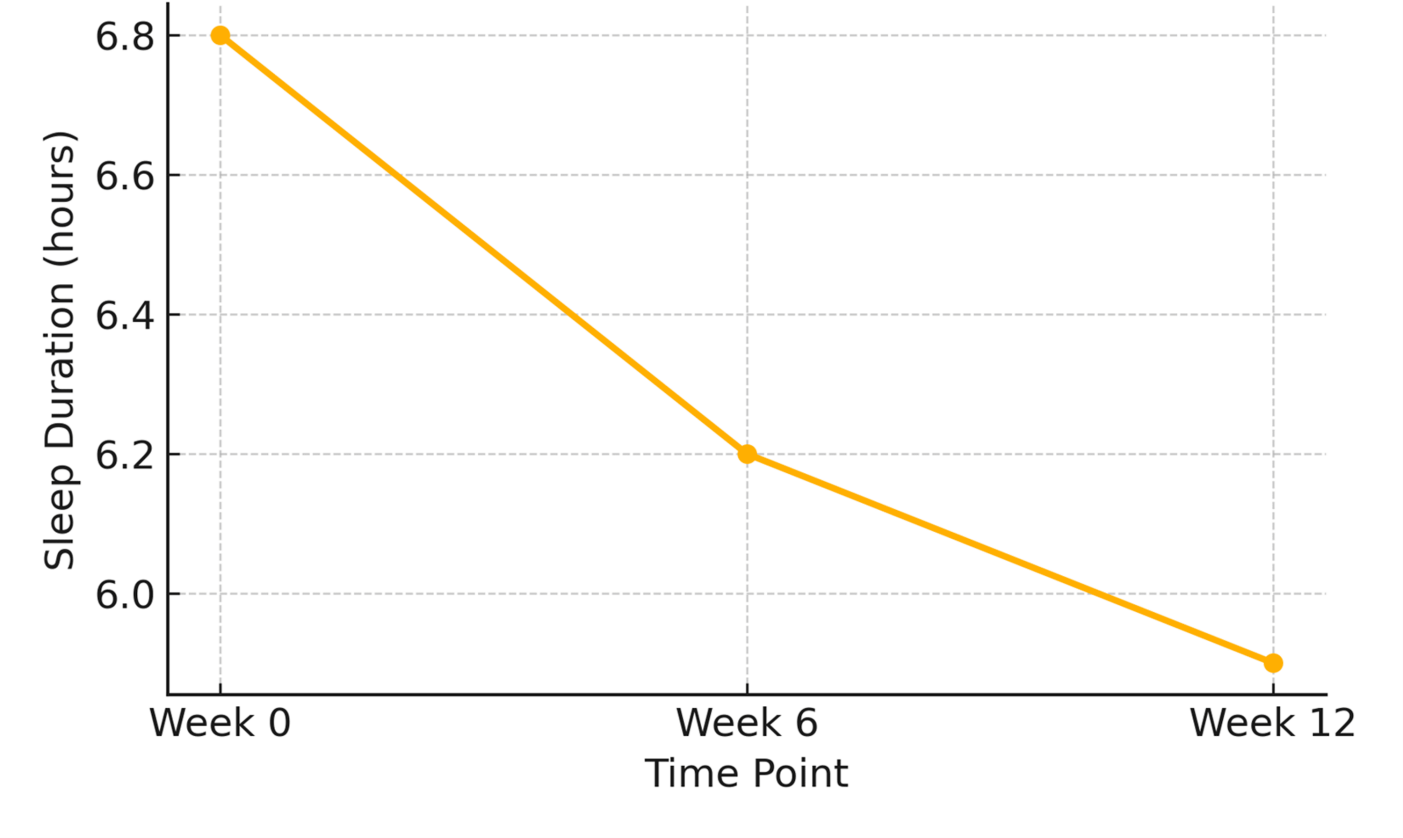
Decline in average sleep duration (hours/night) among medical students at baseline, Week 6, and Week 12 of the study period (n = 72) Statistical test: Repeated-measures ANOVA. F (2,142) = 9.48, p < 0.001. ANOVA: analysis of variance

Over the course of the study, participants reported a significant shift toward poorer sleep quality, with mean Likert scores increasing from 2.1 ± 0.7 at baseline to 3.2 ± 0.9 at Week 12 (p < 0.01). Although 3.2 falls within the neutral range of the scale, the change from baseline represents a meaningful relative deterioration, indicating more frequent sleep disturbances compared to the start of the study. The reduction in the average nightly sleep duration over the 12 weeks is depicted in Figure 2.

Cognitive function changes

Attention (Simple Reaction Time Test)

**Figure 3:**
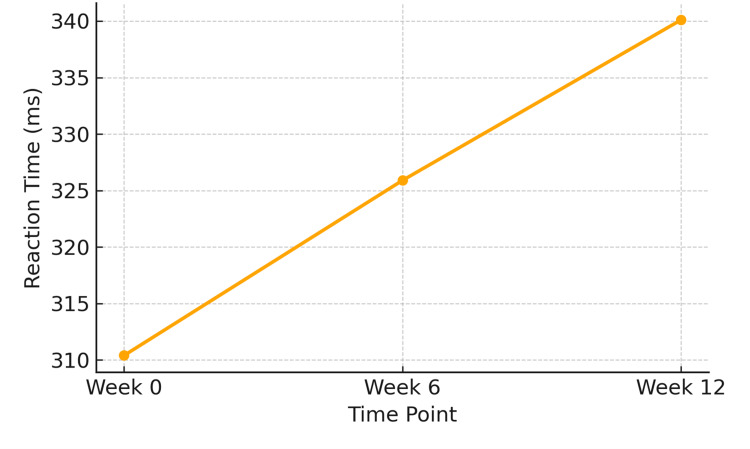
Progressive increase in simple reaction time (ms), indicating reduced attentional efficiency from baseline to Week 12 (n = 72) Statistical test: Repeated-measures ANOVA. F (2,142) = 10.22, p < 0.001. ANOVA: analysis of variance

Simple reaction time increased progressively across the 12-week period, indicating reduced attentional vigilance. Mean reaction time rose from 310.4 ± 25.7 ms at baseline (Week 0) to 325.9 ± 28.2 ms at Week 6, and further to 340.1 ± 30.4 ms at Week 12. This trend was statistically significant (F (2,142) = 10.22, p < 0.001), reflecting a consistent decline in alertness over time (Figure 3).

Working Memory (Digit Span Test)

**Figure 4:**
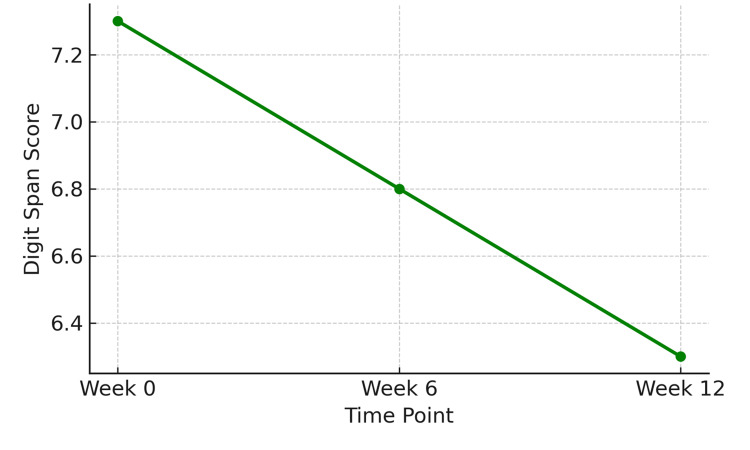
Decline in digit span scores (total) across three time points, reflecting reduced working memory performance over the 12-week period (n = 72) Statistical test: Repeated-measures ANOVA. F (2,142) = 6.71, p = 0.002. ANOVA: analysis of variance

Working memory performance, assessed using the combined total score from Digit Span Forward and Backward formats, declined significantly over the 12‑week period. Mean total scores decreased from 7.3 ± 1.2 at baseline to 6.8 ± 1.1 at Week 6 and further to 6.3 ± 1.0 at Week 12. This decline was statistically significant (F (2,142) = 6.71, p = 0.002), as shown in Figure 4.

Executive Function (Stroop Interference Test)

**Figure 5:**
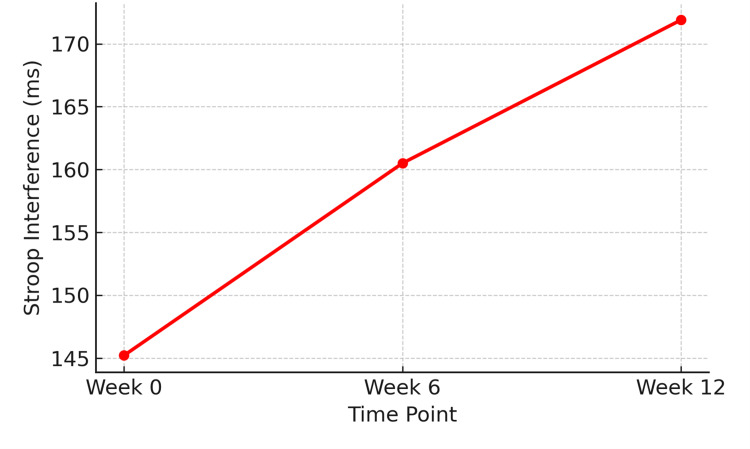
Increase in Stroop interference scores (ms), demonstrating deterioration in executive function over time (n = 72) Statistical test: Repeated-measures ANOVA. F (2,142) = 8.89, p < 0.001. ANOVA: analysis of variance

Executive function, measured by Stroop interference time, worsened progressively over the study period. Mean Stroop scores increased from 145.2 ± 20.3 ms at baseline to 160.5 ± 22.1 ms at Week 6 and 171.9 ± 23.6 ms at Week 12. This increase was statistically significant (F (2,142) = 8.89, p < 0.001), indicating a decline in executive control abilities (Figure 5).

Academic performance

Final academic performance scores differed significantly across sleep groups. Students in the high sleep group (> 6.5 hours) scored 69.4 ± 8.3%, while those in the moderate (5.5-6.5 hours) and low sleep groups (< 5.5 hours) scored 64.8 ± 7.5% and 59.2 ± 6.9%, respectively (F (2,69) = 7.18, p = 0.001). This trend suggests a dose‑dependent positive association between sleep duration and academic achievement, indicating that despite greater academic load often being associated with reduced sleep time, students who maintained longer sleep durations achieved better scores.

**Figure 6:**
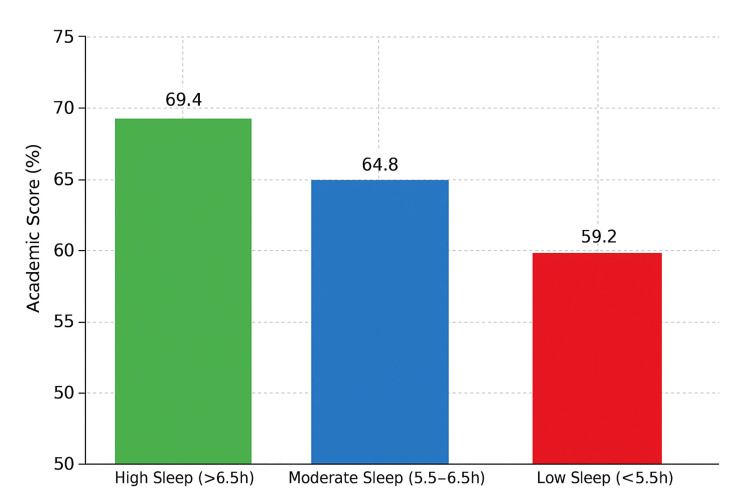
Comparison of academic performance (% scores) among three groups stratified by average sleep duration: high, moderate, and low sleep categories (n = 72) Statistical test: One-way ANOVA. p < 0.01. ANOVA: analysis of variance

The between-group difference was statistically significant. Figure 6 presents a comparison of academic performance across different sleep duration categories.

Confounder descriptive statistics

At baseline, the mean screen time was 5.6 ± 1.4 hours/day; 41 (57%) participants reported screen use within one hour of bedtime. The mean PSS score was 19.7 ± 5.2, with 45 (62%) students classified as experiencing moderate stress. Chronotype distribution showed 22 (31%) were morning types, 35 (48%) were intermediate, and 15 (21%) were evening types. Additionally, 11 (14%) students were excluded due to moderate-to-severe anxiety or depression, as per the DASS-21 screening.

Table 2 presents the Pearson correlation matrix, illustrating the interrelationships between sleep duration, cognitive test scores, and academic performance prior to multivariable adjustment.

**Table 2:** Pearson correlation coefficients (r) and two‑tailed p‑values between sleep duration, cognitive test scores, and academic performance (n = 61*) Pearson’s r values indicate strength and direction of bivariate associations (positive = direct, negative = inverse). p‑values are two‑tailed; statistical significance at p < 0.05. *n = 61 after exclusion of participants with moderate-to-severe anxiety or depression (DASS‑21). DASS-21: Depression, Anxiety, and Stress Scale-21

Variable	Sleep duration (r, p)	Reaction time (r, p)	Digit span (r, p)	Stroop score (r, p)	Academic score (r, p)
Sleep duration	1.00	-0.47 (p < 0.001)	0.39 (p = 0.002)	-0.41 (p = 0.001)	0.43 (p = 0.001)
Reaction time	-0.47 (p < 0.001)	1.00	-0.36 (p = 0.005)	0.49 (p < 0.001)	-0.44 (p = 0.001)
Digit span	0.39 (p = 0.002)	-0.36 (p = 0.005)	1.00	-0.33 (p = 0.009)	0.40 (p = 0.002)
Stroop score	-0.41 (p = 0.001)	0.49 (p < 0.001)	-0.33 (p = 0.009)	1.00	-0.38 (p = 0.004)
Academic score	0.43 (p = 0.001)	-0.44 (p = 0.001)	0.40 (p = 0.002)	-0.38 (p = 0.004)	1.00

Multivariable analyses

Academic Performance Prediction

Multivariable linear regression revealed that sleep duration was a significant independent predictor of academic performance (β = +2.78, p = 0.003), even after adjusting for screen time (β = -1.41, p = 0.005), perceived stress (β = -0.53, p = 0.002), and evening chronotype (β = -2.93, p = 0.007). The overall model was significant (F (5,66) = 8.14, p < 0.001) with an adjusted R² of 0.38, indicating moderate explanatory power. Table 3 displays the results of the multivariable model examining sleep duration and confounders as predictors of academic performance.

**Table 3:** Multivariate regression predicting academic performance (n = 72) Model summary: Adjusted R² = 0.38; F (5,66) = 8.14, p < 0.001 β: regression coefficient; CI: confidence interval; R²: coefficient of determination; F: model F-statistic; PSS: Perceived Stress Scale

Predictor	β coefficient (95% CI)	p-value
Sleep duration	+2.78 (1.06 – 4.51)	0.003
Screen time	-1.41 (-2.37 – -0.45)	0.005
PSS score	-0.53 (-0.87 – -0.19)	0.002
Diet (poor vs healthy)	-1.85	0.082
Evening chronotype	-2.93 (-5.02 – -0.84)	0.007

Working Memory Prediction

In the multivariable model predicting working memory, sleep duration was positively associated with digit span scores (β = +0.29, p = 0.010), while perceived stress (β = -0.13, p = 0.016) and screen time (β = -0.18, p = 0.031) were negatively associated. The model explained 26% of the variance (R² = 0.26, p < 0.01). Table 4 summarizes the multivariable analysis identifying sleep and stress as independent predictors of working memory (digit span). 

**Table 4:** Multivariate regression predicting digit span score (n = 72) Model summary: R² = 0.26, p < 0.01. β: regression coefficient; R²: coefficient of determination; PSS: Perceived Stress Scale

Predictor	β coefficient (p-value)
Sleep duration	+0.29 (0.010)
PSS score	-0.13 (0.016)
Screen time	-0.18 (0.031)

Model diagnostics

All regression assumptions were verified prior to interpretation. Multicollinearity was minimal, with all VIF values < 2.1. Residuals were normally distributed, confirmed via Shapiro-Wilk tests (p > 0.20 for all models). No significant interaction was found between sleep duration and chronotype (p > 0.05), indicating independent effects.

Adjusted correlation summary

After adjusting for confounding variables, sleep duration remained a significant independent predictor across all outcome domains. It was positively associated with academic performance (β = +2.78, p = 0.003) and digit span (β = +0.29, p = 0.010) and inversely associated with reaction time (β = -4.12, p = 0.015) and Stroop interference (β = -3.85, p = 0.021), reflecting improved attention and executive function with greater sleep duration. A comprehensive summary of the adjusted associations between sleep and each outcome is presented in Table 5.

**Table 5:** Confounder-adjusted correlation summary between sleep and outcome variables (n = 72) Model summary: Adjusted R² = 0.38; F (5,66) = 8.14; p < 0.001. β: regression coefficient; R²: coefficient of determination; F: ANOVA test statistic; ANOVA: analysis of variance

Outcome variable	Adjusted β (sleep duration)	p-value
Academic performance (%)	+2.78	0.003
Digit span (working memory)	+0.29	0.010
Reaction time (attention)	-4.12	0.015
Stroop interference (executive function)	-3.85	0.021

## Discussion

Principal findings

This longitudinal study presents compelling evidence that prolonged sleep deprivation among medical undergraduates leads to a progressive decline in critical cognitive domains, specifically attention, working memory, and executive function, as well as a noticeable decline in academic performance. These effects remained significant even after controlling for behavioral and psychological confounders, underscoring the independent role of sleep in maintaining neurocognitive and academic functions. To the best of our knowledge, this is among the first longitudinal Indian studies directly linking sleep decline to cognitive and academic metrics over a structured academic phase.

Sleep Deprivation and Student Life: A Hidden Curriculum

The documented reduction in sleep duration - from 6.8 to 5.9 hours per night - aligns with previous findings that medical students frequently do not meet recommended sleep standards [2,3]. This pattern likely results from a combination of academic demands, irregular schedules, and insufficient awareness of sleep hygiene, particularly during the preclinical stage. Our findings indicate not only a quantitative reduction in sleep, but also a qualitative deterioration, as evidenced by increased subjective reports of poor sleep quality [4]. Based on these findings, we recommend that medical education programs incorporate structured wellness and sleep‑hygiene modules into the curriculum. Such initiatives could include time‑management and stress‑reduction workshops, chronotype‑informed scheduling, and counseling services aimed at promoting healthy sleep habits. Implementing these strategies may help preserve cognitive performance and optimize academic outcomes for medical students.

Trajectory of Cognitive Impairment

Increasing reaction times indicate a reduction in attentional vigilance, likely reflecting the accumulation of central fatigue [15]. Deterioration in digit span scores suggests a disruption in working memory, which is essential for encoding and integrating new information. Similarly, an increase in Stroop interference signifies a weakening of executive control, which is crucial for prioritization, decision-making, and error management. These observations are consistent with the neurobiological literature that associates sleep deprivation with hypoactivation of the prefrontal cortex and altered hippocampal connectivity, which in turn impairs attentional and memory networks [16,17]. Sleep facilitates cognitive function through several mechanisms, including hippocampo-cortical memory consolidation during slow-wave sleep, synaptic homeostasis, and the recalibration of executive functions via restored prefrontal activity [18]. The cognitive decline observed in our cohort likely reflects the disruption of these restorative pathways.

Confounder-Controlled Clarity

This study addresses the methodological limitations of previous research by incorporating validated instruments to evaluate screen time, dietary habits, psychological stress, and circadian preference (chronotype). After controlling for these variables using multivariate regression models, sleep duration emerged as a significant independent predictor of both cognitive test performance and academic outcomes. Sleep is not merely a correlation of performance but rather a potentially modifiable determinant. Excessive screen exposure, particularly during nighttime, may delay the onset of melatonin and suppress rapid eye movement (REM) sleep, thereby elucidating its adverse association with cognitive performance [19]. Additionally, elevated stress levels are correlated with poorer outcomes, likely mediated by cortisol-induced suppression of the hippocampus. While these confounding factors are significant, they do not overshadow the predominant effect of sleep duration [20].

Notably, students exhibiting an evening chronotype demonstrated inferior academic performance, even when the quantity of sleep was comparable. This observation underscores the significance of circadian misalignment. It aligns with emerging chronobiological research that advocates personalized academic scheduling based on chronotypes to enhance learning outcomes [21].

Educational Implications

This study highlights the necessity of reconceptualizing sleep as an essential academic competency rather than merely passive rest. Educators should consider implementing interventions such as time management training, sleep hygiene workshops, and chronotype-informed scheduling to enhance academic environments. Additionally, institutions ought to reassess clustered assessments, which reduce sleep opportunities and increase performance pressure.

Strengths and limitations

This study possesses several notable strengths. Foremost, its longitudinal design, incorporating repeated assessments at multiple time points, facilitates temporal inferences concerning the decline in sleep and cognitive performance. Second, cognitive outcomes were assessed through the use of standardized, computerized testing protocols, thereby enhancing the objectivity of the measurements. Third, the study accounted for essential confounding variables, including stress, screen time, chronotype, and diet, utilizing validated instruments. Finally, the high participant retention rate (> 90%) effectively minimized attrition bias and enhanced the reliability of the data.

Nevertheless, certain limitations of the study must be acknowledged. As academic performance was based solely on physiology assessments, it may not fully reflect students’ overall academic abilities or subject-specific strengths in other areas. The sleep decline coincided with the academic term but may also reflect lifestyle, psychosocial, or environmental factors. The research did not utilize objective sleep-tracking devices, such as actigraphy, and instead relied on self-reported sleep diaries. While diaries offer valuable longitudinal data, the use of standardized and validated self-report sleep questionnaires (e.g., Pittsburgh Sleep Quality Index [22] or Epworth Sleepiness Scale [23]) could have enhanced the reliability and comparability of subjective sleep measures. Self-reported sleep duration, though practical and feasible for large cohorts, has moderate validity compared to objective measures and often overestimates actual sleep by 30 to 60 minutes. Despite these biases, diaries reliably detect relative differences and temporal changes in sleep patterns, particularly in healthy student populations. In our study, the observed longitudinal trends and their associations with cognitive and academic outcomes remain meaningful, but future research combining self-reports with objective sleep monitoring may further enhance precision. Moreover, several potentially influential variables, including academic motivation, personality traits, and social support, were not assessed and may have contributed to the variability in outcomes. Changes in anthropometric measures, particularly BMI, and socio‑demographic factors such as socioeconomic status, parental education, and residential background can influence both cognitive function and academic performance. Higher BMI has been linked with reduced executive function and academic achievement in some studies, while socio‑demographic factors often shape learning environments, lifestyle behaviors, and access to resources. Although these variables were not assessed in the present study, future research should consider them as potential confounders when examining the relationship between sleep, cognition, and academic outcomes. Lastly, the study's single-center design may constrain its applicability to a wider population of medical students.

Future studies using wearable technology and multicentric designs are required to validate and extend these findings.

## Conclusions

This longitudinal study found that chronic sleep deprivation among Indian medical undergraduates is strongly associated with progressive declines in attention, working memory, executive function, and academic performance. These effects remained significant even after adjusting for stress, screen time, diet, and chronotype, underscoring sleep duration as an independent determinant of cognitive and academic outcomes.

## Appendices

Appendix A

**Table 6:** Academic Motivation Profile Questionnaire (AMPQ) Response Scale: 1 = Strongly Disagree, 2 = Disagree, 3 = Neutral, 4 = Agree, 5 = Strongly Agree. Scoring: Domain-wise summation or averaging of item scores. PG: postgraduate Credits: The questionnaire was developed by the authors.

Domain	Item	Response format
Intrinsic motivation	I study because I find the topics personally interesting.	1–5 Likert Scale
	I enjoy learning new concepts even if they are not part of the exam.	1–5 Likert Scale
	I feel satisfied when I solve difficult academic problems.	1–5 Likert Scale
Extrinsic motivation	I study to achieve good grades and stand out academically.	1–5 Likert Scale
	I put in effort so that my family/peers see me as successful.	1–5 Likert Scale
	I study to qualify for future opportunities (e.g., PG entrance).	1–5 Likert Scale
Amotivation	I often question why I am studying medicine at all.	1–5 Likert Scale
	I sometimes feel that studying doesn’t really improve my understanding.	1–5 Likert Scale
	I study only because I have no choice, not because I want to.	1–5 Likert Scale

Appendix B 

**Table 7:** Food Frequency Questionnaire (FFQ) Instructions: Indicate how frequently you consumed the following items in the past seven days. Scoring (if required): Frequency scores can be numerically weighted for trend analysis. Credits: The questionnaire was developed by the authors.

No.	Food item	Frequency options
1	Milk and dairy products	Never / 1–2 times / 3–4 times / Daily
2	Fruits	Never / 1–2 times / 3–4 times / Daily
3	Green leafy vegetables	Never / 1–2 times / 3–4 times / Daily
4	Fast food (e.g., burgers, pizza)	Never / 1–2 times / 3–4 times / Daily
5	Caffeinated beverages (tea, coffee, energy drinks)	Never / 1–2 times / 3–4 times / Daily
6	Soft drinks/sugary beverages	Never / 1–2 times / 3–4 times / Daily
7	Fried snacks (e.g., samosas, pakoras)	Never / 1–2 times / 3–4 times / Daily
8	Whole grains (e.g., brown rice, whole wheat bread)	Never / 1–2 times / 3–4 times / Daily
9	Non-vegetarian foods (chicken, fish, eggs)	Never / 1–2 times / 3–4 times / Daily
10	Sugary items (sweets, chocolates)	Never / 1–2 times / 3–4 times / Daily
